# Distinct Genetic Influences on Cortical and Subcortical Brain Structures

**DOI:** 10.1038/srep32760

**Published:** 2016-09-06

**Authors:** Wei Wen, Anbupalam Thalamuthu, Karen A. Mather, Wanlin Zhu, Jiyang Jiang, Pierre Lafaye de Micheaux, Margaret J. Wright, David Ames, Perminder S. Sachdev

**Affiliations:** 1 Centre for Healthy Brain Ageing, School of Psychiatry, University of New South Wales, Sydney, NSW 2052, Australia; 2 Neuropsychiatric Institute, Prince of Wales Hospital, Randwick, NSW 2031, Australia; 3 Department of Mathematics and Statistics, University of Montreal, Montreal, QC H3T1J4, Canada; 4 School of Mathematics and Statistics, University of New South Wales, Sydney, NSW 2052, Australia; 5 Queensland Brain Institute, University of Queensland, St Lucia, QLD 4072, Australia; 6 Centre for Advanced Imaging, University of Queensland, St Lucia, QLD 4072, Australia; 7 National Aging Research Institute, University of Melbourne, Parkville, VIC 3052, Australia

## Abstract

This study examined the heritability of brain grey matter structures in a subsample of older adult twins (93 MZ and 68 DZ twin pairs; mean age 70 years) from the Older Australian Twins Study. The heritability estimates of subcortical regions ranged from 0.41 (amygdala) to 0.73 (hippocampus), and of cortical regions, from 0.55 (parietal lobe) to 0.78 (frontal lobe). Corresponding structures in the two hemispheres were influenced by the same genetic factors and high genetic correlations were observed between the two hemispheric regions. There were three genetically correlated clusters, comprising (i) the cortical lobes (frontal, temporal, parietal and occipital lobes); (ii) the basal ganglia (caudate, putamen and pallidum) with weak genetic correlations with cortical lobes, and (iii) the amygdala, hippocampus, thalamus and nucleus accumbens grouped together, which genetically correlated with both basal ganglia and cortical lobes, albeit relatively weakly. Our study demonstrates a complex but patterned and clustered genetic architecture of the human brain, with divergent genetic determinants of cortical and subcortical structures, in particular the basal ganglia.

Brain structure is under strong genetic control, but how genes influence the organization of the brain is only beginning to be understood^1^. The patterning of the cerebral cortex, known as arealization, has been systematically studied in animals^2^,^3^. Work on transcription factors, morphogens and signalling molecules in the rodent has shown how the regional identity of cortical areas develops^4^. The neocortex is also massively connected to the thalamus, basal ganglia and the hippocampus, but the genetic basis of specialisation of the subcortical nuclei and their relationship to cortical arealisation has received much less attention^1^. Twin studies have shown that the volumes of subcortical structures are under strong genetic control which accounts for 50–80% of the variance^5^,^6^,^7^,^8^. A recent collaborative study has identified common genetic variants that influence the volumes of the hippocampus, putamen and caudate^9^, but each accounted for only a small proportion of the heritability (h^2^). In the search for genes related to brain structures, it is important to know whether the relevant genes are shared by different structures or are unique to each structure. Additionally, cortical and subcortical structures are structurally and functionally related, and activity in one can help pattern the other. For instance, thalamocortical afferents are important in the ‘extrinsic’ patterning of the cortex through thalamic input^4^. While genetic influence on human cortex was previously investigated^10^,^11^, whether and how the genetic influence on cortical and subcortical structures is shared is an intriguing question that to date has not been examined.

The adult brain undergoes structural and functional changes with ageing, and these age-related changes are influenced by both genetic and environmental factors. The regional patterning of the older brain is therefore likely to be the result of a complex interplay of development and ageing-related genes and interaction with extrinsic factors. Furthermore, developmental and lifespan changes in cortical thickness was found to associate with the underlying genetic organizational principles of cortical thickness^12^. Unravelling this complexity is important to understand some of the mechanisms that underlie age-related brain diseases^13^.

The study of monozygotic (MZ) and dizygotic (DZ) twins permits the estimation of heritability of a particular phenotypic measure, which is the proportion of the phenotypic variance accounted for by genetic factors. Twin studies of the cortex and subcortical structures using neuroimaging phenotypes have shown strong genetic influences on the volumes of these structures^7^,^14^,^15^,^16^. Multivariate methods can be applied to twin data to examine the genetic relationships between multiple phenotypes, informing us whether common or specific genetic influences are at work. Previous research on the genetic organization of the human brain has examined the cortical surface area and cortical thickness in men^17^,^18^,^19^, and the genetic contributions to subcortical structures and their correlations in young^5^,^8^ and middle aged individuals^6^. To further our understanding of the genetic patterning of the human brain, the main objective of our study was to examine the genetic relatedness of cortical and subcortical structures in the Older Australian Twins Study, comprising individuals 65 years and older.

We used the grey matter volumes in our study and both cortical and subcortical structures were extracted using FreeSurfer^20^. The cortex was segmented into thirty-four gyral regions per hemisphere based on the Desikan-Killiany Atlas^21^. We calculated the volumes of four major lobes, i.e. frontal, occipital, parietal, and temporal as the sum of the relevant ROIs to reduce the number of parameters for some of the univariate and multivariate genetic modelling. The volumes of seven subcortical structures thalamus, caudate, putamen, pallidum, hippocampus, amygdala, and nucleus accumbens, and the Intracranial volume (ICV) were also measured. Heritability and genetic correlations adjusted for age, sex, scanners and ICV were estimated using univariate and multivariate structural equation modelling (SEM)^22^. Additional technical details on univariate and multivariate genetic modelling used here can be found in the methods section together with Figs 1 and 2.

## Results

The descriptive statistics of our samples are given in Table 1. The sample consisted of 322 participants without dementia, comprising 93 MZ and 68 DZ (25 opposite sex) twin pairs, all Caucasian, with a mean age of 70.1 (±4.9) years (range 65–85), 11.04 (±3.3) years of education, and a Mini-Mental State Examination (MMSE)^23^ score of 28.6 (±1.69).

### Heritability of cortical and subcortical structures

The heritability estimates for total (left plus right hemisphere) volumes of the subcortical regions ranged from 0.41 for the amygdala to 0.73 for the hippocampus (see Table 2). For the cortical lobar regions, heritability ranged from 0.55 for the parietal lobe to 0.78 for the frontal lobe. The total intracranial volume (ICV) was highly heritable (h^2^ = 0.79). More finely defined individual cortical ROIs (region of interest) based on the Desikan-Killiany atlas^21^ showed varying levels of heritability, ranging from ~0 (e.g. caudal anterior-cingulate cortex) to 0.67 (e.g. precentral gyrus, insula cortex). In general most of the small individual ROIs have very low heritability estimates with wider confidence intervals. Both cerebral hemispheres showed similar levels of heritability (Supplementary Table S1).

### Bilateral symmetry of cortical and subcortical volumes

The heritabilities of the corresponding ROIs of the two hemispheres were found to be similar. To compute genetic correlations and test the significance of bilateral genetic sharing an Independent Pathway Model (IPM)^22^ was used (see the methods section for details). The genetic correlations between the ROIs of two hemispheres were found to be high (>0.7), suggesting common genetic determinants for the two hemispheres. The genetic contribution of the common factor of the two ROIs was greater than the specific genetic variance. The specific environmental variances were higher than the common environmental variance. The genetic correlations were much higher than the environmental correlations (Supplementary Table S2). For most of the ROIs, the genetic correlations between the homologous regions were found to be highly significant and also the test of genetic correlations r_G_ = 1 tenable. The ROIs of caudal anterior cingulate, cuneus cortex, parahippocampal gyrus, rostral middle frontal gyrus, superior frontal gyrus, insula cortex, thalamus, pallidum and amygdala, the specific genetic components of the left and right regions were found to be different and hence r_G_ were not unity.

### Genetic overlap between cortical lobar and subcortical volumes

We evaluated the genetic correlations among different ROIs (total volume as the sum of left and right) of the whole brain. The heatmap of genetic correlations among all the ROIs is presented in Supplementary Figure S1. The genetic correlations between cortical gyral ROIs again varied considerably. In general, genetic correlations were higher between ROIs within the same lobe than between ROIs in different lobes, and the genetic correlations between subcortical regions and cortical ROIs were low.

Central to the aim of our study was the genetic sharing of the whole brain grey matter, including cortex and subcortical grey matter structures. A multivariate SEM was used to study the patterns of genetic correlations among the four cortical lobes and seven subcortical regions. Starting from the saturated Cholesky model^22^ together with the factor analysis, we identified a parsimonious three factor IPM with a single shared environmental component (Fig. 2). The first two factors divided the subcortical regions into two groups and the third factor corresponded to the cortical lobes. The first factor explained 19.6% of total genetic variance (sum of squares of path coefficients corresponding to a_c1_ divided by the sum of squares of path coefficients corresponding to a_c1_, a_c2_, a_c3_ and the eleven specific genetic components) with the majority (89%) explained by the hippocampus, amygdala and thalamus (total genetic variance of a_c1_ was calculated as sum of squares of its path-coefficients), compared to 9.5% attributed to the cortical lobes. Similarly, the second factor explained 15.8% of the total genetic variance which represented the caudate, pallidum and putamen and the third factor explained 13.2% of genetic variance and included the four cortical lobes.

We observed that genetic and environmental correlations between cortical lobes had similar magnitude, with the highest genetic correlation observed between the parietal and temporal lobes (Table 3). In general, environmental correlations between subcortical structures were higher than their corresponding genetic correlations; indeed, some genetic correlations were negative. A similar pattern was observed between subcortical structures and cortical lobes, again with higher environmental than genetic correlations, and some negative genetic correlations. It should be however noted that genetic clusters use only the genetic correlations and hence clusters of ROIs are solely dependent on the extent of genetic correlations and not on the magnitude of environmental correlations.

The cluster analysis of the genetic correlation matrix based on the three-factor IPM also demonstrated three distinct clusters. Similar clustering was observed based on the phenotype correlation matrix obtained using the three factor IPM factor analysis (Fig. 3).

## Discussion

We report a comprehensive examination of the genetic influence on cortical and subcortical structures in older adults of both sexes. In general, cortical and subcortical structures had moderate to high heritability, suggesting a strong underlying genetic basis for these brain phenotypes. Our analyses examined human cortex and subcortical structures together and showed that in the context of whole brain grey matter structures, cortical lobes were genetically correlated with each other and formed a genetic cluster. The basal ganglia structures were highly correlated among themselves, forming a second cluster, and the amygdala, hippocampus, thalamus and nucleus accumbens formed a third cluster. The hierarchical division of the eleven structures into three clusters demonstrated fundamentally different genetic associations for cortical regions, basal ganglia and the other subcortical structures that are functionally and anatomically close to each other.

The fact that our study demonstrated that human brain cortex genetically formed a cluster of its own and the cortex was only weakly correlated to subcortical structures provided good evidence for investigating the genetics of these two divisions of cerebral grey matter independently. The vast majority of the literatures so far have indeed examined human brain cortex^10^,^11^,^17^,^18^,^19^,^24^,^25^,^26^,^27^ or subcortical structures^5^,^6^,^8^,^9^,^28^ separately and our findings are largely consistent with the literature in both cortical lobes and subcortical structures.

Our heritability estimates were moderate to high for both cortical and subcortical structures as has been previously reported^14^,^15^,^16^, but considerable heterogeneity in heritability values was observed. Of the subcortical structures, the hippocampus, putamen and caudate yielded higher heritability estimates, although confidence intervals overlap for most of the subcortical structures. Compared to other subcortical regions, the heritability estimates were lower for amygdala and nucleus accumbens, which is in agreement with previous reports^5^,^8^,^25^.

Structures in the two hemispheres showed similar levels of heritability, and modelling showed a common genetic factor accounting for much of the heritability in corresponding regions bilaterally. This is again consistent with previous reports which showed that the two hemispheres were genetic mirror images of each other^7^,^17^,^18^,^19^.

A previous study has shown that the influences of several common genetic polymorphisms on the brain are region specific and bilateral^29^. The aggregate effects of many polymorphisms such as this could explain the patterns of bilateral similarity. This is further supported by data on gene expression patterns, which are also mirrored in the two hemispheres^30^. Such lateralization is also reflected in the anatomical patterns of bilateral atrophy in both normal ageing^31^ and many neurodegenerative syndromes such as Alzheimer’s disease^32^. Since the evidence of bilateral symmetry comes from an older cohort used in our study, it is consistent with bilateral symmetry for genetic influences on brain development as well as age-related change.

The most noteworthy finding of this study is the distinct genetic influences on cortical and subcortical structures, with little genetic covariation between any of the four cortical lobes and the seven subcortical regions. In our study, the shared genetic variance for the cortical regions, to some extent, had a lobar pattern, with regions within a lobe having higher genetic correlations (Supplementary Figure S1). For the lobes, the genetic similarity was greatest for the temporal, parietal and frontal lobes, with the occipital lobe showing lower shared genetic variance. Our data are consistent with those reported previously^17^,^18^, considering that our imaging measures were volumes which were influenced by both cortical area and thickness. Our approach of using pre-defined anatomical regions could not examine genetic variance according to functional specialisation in the cortex, but the clustering of lobar regions is a noteworthy pattern.

The basal ganglia (caudate, putamen and pallidum) formed a genetic cluster and the parts of the basal ganglia had either no genetic correlation (pallidum, putamen) or a weak but negative one (caudate) with cortical volume measures. The cluster comprising the amygdala, hippocampus, and thalamus had moderate genetic correlations with the cortical regions, and the correlation between hippocampus and temporal lobe was relatively strong. Within the cluster that comprised the amygdala, hippocampus, nucleus accumbens and thalamus, the amygdala and hippocampus had a high genetic correlation between them, and both structures, along with the nucleus accumbens, had moderate genetic correlations with the thalamus.

The correlations were estimated with ICV as a covariate. This is because a major proportion of the shared genetic variance between different brain regions is explained by a common genetic factor which also accounts for head size, and its proxy, the ICV^5^. It has been noted that some literature^33^,^34^ found that head size was related to body height, suggesting that general growth pathways influence both body and brain development which include the growth hormone and insulin-like growth factor 1^35^,^36^,^37^. We also note that there was report^38^ which did not share the same results.

The regionalisation of the brain occurs early in development and the precursor of both cortical and subcortical structures is the prosencephalon, which divides into the telencephalon and diencephalon^39^. The diencephalon gives rise to the thalamus and hypothalamus, and the telecephalon or the forebrain is the precursor of the cortex and subcortical structures such as the basal ganglia, hippocampus and amygdala. This patterning of the nervous system is under the control of various signalling pathways, such as sonic hedgehog, Wnt, retinoids, fibroblast growth factors and transforming growth factor-β^40^. Both extrinsic and intrinsic factors have been identified that interact to develop specific telencephalic domains^41^. It is not yet understood how these multiple patterning factors regulate each other’s function, and there are certainly other factors that are not known. Our study shows that genetic factors play a major role in this patterning, with common genetic factors shaping some subcortical structures but not others.

The human brain distinguishes from that of other species with the enormous expansion of the neocortex relative to total brain volume. With evolution, cortical thickness increases together with more dramatic enlargement in cortical surface size,^42^ which contribute to cortical volumes that we used as the imaging phenotype in this study. The relative genetic homogeneity between the cortical lobes in comparison with subcortical structures may reflect the similarity in laminar architecture across the entire neocortex^43^. It was found that there was a remarkable degree of transcriptional uniformity of cortex compared to other brain regions^30^.

For the subcortical structures, Our findings suggest that the volumes and sizes of cortex and subcortical structures in ageing stages are not determined by a single set of genes, which are consistent with previous reports, but with a few notable differences. A previous study of middle aged male twins^6^ identified a basal ganglia/thalamic factor and a limbic factor (hippocampus and amygdala) for shared genetic influence. A study of younger twins^5^ identified four factors: basal ganglia (caudate, putamen and pallidum), nucleus accumbens, amygdala and hippocampus/thalamus. Our results are closer to the latter study, although the genetic correlations between nucleus accumbens and other structures of the cluster were low, being 0.11 with amygdala, 0.21 with hippocampus and 0.10 with thalamus in our study, after accounting for ICV. It is important to emphasize the consistencies between our findings and the literature. This is because although all three studies used the volumetrics extracted by FreeSurfer, ours had controlled for ICV and the young adult twins study^5^ and middle-aged male^6^ study had not. Despite the large differences in ages of the samples or whether the sample consisted both sexes or males only, one of the common findings of previous studies^5^,^6^ and ours was that nucleus accumbens had the lowest heritability as well as relatively low genetic correlation with o ther subcortical structures and with cortical lobes as shown in our sample. We speculate that one of the reasons that heritability values of nucleus accumbens and amygdala being relatively low was because of the less accurate segmentation due to the difficulties in delineation of a clear boundary. The relatively large variances because of their smaller sizes in comparison with other subcortical structures may also contribute to their weaker genetic correlations and thus large environmental factors^5^,^6^,^7^.

The OATS cohort had mean age of 70 years (range 65–85), and one would expect that the volumes of brain structures at this age are an end result of early developmental and later degenerative changes. Age-related changes in cortical thickness were found to follow closely the genetic organization of the cerebral cortex, and genetic factors contributed to cortical changes through life^12^. Our study has not examined the atrophy of the sample. An unexplored question is the interactions between genetics and brain diseases and how the trajectories of changes, such as how cortical and subcortical atrophy would differ from the normal genetic organization in different brain diseases. In an imaging study including both Alzheimer’s Disease (AD) and behavioural-variant frontotemporal dementia patients, it was reported^44^ that AD patients had greater cortical atrophy than behavioural-variant frontotemporal dementia patients while behavioural-variant frontotemporal dementia patients had greater atrophy in subcortical regions, especially in the striatum than AD patients over time. Indeed, our findings suggest that cortical and subcortical volumetrics are weakly related genetically and age-related changes to these structures may take distinctively different trajectories. However, how genetic architecture and disease interplay and impact on their ageing and pathological trajectories are complex and future investigations should take into account of both. MRI studies show that cortical thickness starts to decrease from childhood^45^,^46^, and the age-related trajectories are often nonlinear such as observed in the basal ganglia^47^. Our work focused on the ageing population as the mean age of our participants were 70 and there is evidence of an age-related reduction in volume, which accelerates with age^31^,^48^. The age-related changes have been well documented for the hippocampus^49^. It is quite likely that the genetic influences of neurodevelopment and patterning are different from the genes that determine age-related decline. One would expect that the genetic contributions to brain structure would be higher in the younger cohort and environmental factors would become more prominent with ageing. Data from cross-sectional studies support this conclusion^15^.

Our study has a number of limitations. First, we subdivided the cortex using a non-genetically based atlas^21^ which uses major sulci for lobar and regional classification, and the subcortical structures were delineated by their anatomical boundaries. Since each cortical parcellation and/or lobe comprises smaller sub-entities that may be genetically heterogeneous^18^, our approach to use non-genetically based cortical subdivision may bring in unintended inaccuracy. Similarly, the assumption that a subcortical structure such as the caudate is genetically homogenous is unlikely to be true. Ideally, the genetic basis of these structures should be examined with no anatomical constraints, but this introduces a higher level of complexity in the analysis, which must then proceed structure by structure rather than at the whole brain level. Second, as noted above, segmentation of nucleus accumbens and amygdala may be less accurate than other subcortical structures. As our primary interest is to show only the patterns of heritability and genetic correlations, all our results are presented without correcting for multiple hypotheses testing. Although it was said that surface area and cortical thickness had been found to be genetically and phenotypically independent^50^ and volumes which we used in the present study were the product of these two, the volumetrics had been the better imaging phenotype for our study because we could use volumes for both cortex and subcortical structures and our question was how genetic influence on cortical and subcortical structures was shared.

In conclusion, this study is the first attempt to examine the genetic correlations between human cortex and subcortical structures, using the twin design. The data showed that cortical and subcortical structures had moderate to high heritability, and formed three genetic clusters. The cortical lobes (frontal, temporal, parietal and occipital) were genetically correlated with each other and formed one cluster. The basal ganglia (caudate, putamen and pallidum) were highly correlated with each other, forming a second genetic cluster, and the amygdala, hippocampus, thalamus and nucleus accumbens formed a third cluster. Additionally, the genetic influences on brain structures were bilaterally symmetrical. This patterning of the heritability of brain structures has important implications for investigations into the genetic blueprint of the human brain.

## Methods

### Participants

Our study cohort was drawn from Wave 1 of the Older Australian Twins Study (OATS), a study of twins aged 65 years or older living in the three Eastern states of Australia (New South Wales, Victoria and Queensland) primarily recruited from the Australian Twin Registry (ATR). Methodology of OATS has previously been described in detail^51^. The zygosity of each twin pair had been confirmed previously by genotyping with high-density single nucleotide polymorphism arrays. This study was approved by the ethics committees of the Australian Twin Registry, University of New South Wales, University of Melbourne, Queensland Institute of Medical Research and the South Eastern Sydney & Illawarra Area Health Service. Informed consent was obtained from all subjects and the methods were carried out in accordance with the relevant guidelines.

### Image Acquisition

MRI data were obtained on three 1.5 Tesla scanners and a 3 Tesla scanner owing to the multi-site nature of this study. Siemens Magnetom Avanto and Sonata scanners (Siemens Medical Solutions, Malvern PA, USA) with similar years of manufacture and upgrade were used in centres 2 (Victoria) (114 participants) and 3 (Queensland) (92 participants), respectively. In centre 1 (New South Wales), a 1.5 T Philips Gyroscan scanner (Philips Medical Systems, Best, Netherlands) (80 participants) was used initially, followed by a 3 Tesla Philips Achieva Quasar Dual scanner (36 participants). The acquisition protocols and parameters were tested and matched between the centres through standardization of spatial resolution and slice thickness, using a 3D phantom to correct geometric distortions, and using five volunteers who were scanned on the four scanners^51^. Twin pairs were always scanned on the same scanner and were scanned either on the same day or within a few weeks of each other.

The 3D T1-weighted MRIs scans were used for computing the neuroimaging phenotypes for cerebral cortex and subcortical structures. 3D T1-weighted volumetric sequence was performed using a similar protocol for the 1.5 Tesla scanners in the three centres with in-plane resolution = 1 × 1 mm, slice thickness = 1.5 mm, slice number = 144, TR (Repetition time) = 1530 ms, TE (Echo time) = 3.24 ms, TI (Inversion time) = 780 ms, and flip angle = 8. The acquisition parameters for the 3 Tesla Philips scanner in centre 1 were: TR/TE = 6.39/2.9 ms, in-plane resolution = 1 × 1 mm, slice thickness = 1 mm, slice number = 190, resulting isotropic voxels of 1 × 1 × 1 mm^3^. Two 3D T1-weighted scans were acquired for each participant for an increased signal-to-noise ratio (SNR).

### Image Processing

Scans were excluded if they failed visual quality control. Both cortical and subcortical structures were extracted using FreeSurfer v5.3.0^20^. Briefly, the processing includes motion correction and averaging of the T1-weighted images^52^, removal of non-brain tissue^53^, automated Talairach transformation, segmentation of subcortical structures^54^,^55^, intensity normalization^56^, tessellation of grey matter and white matter boundary and automated topology correction^57^,^58^, and surface deformation^20^. Using Desikan-Killiany Atlas^21^, we segmented cortex into 13 frontal, 9 temporal, 4 occipital, 7 parietal, and insula ROIs and the lobar (four major lobes, i.e. frontal, occipital, parietal, and temporal with both left and right hemisphere) volumes were calculated as the sum of the relevant ROIs to reduce the number of parameters for some of the univariate and multivariate genetic modelling. The volumes of subcortical structures in both left and right hemispheres including thalamus, caudate, putamen, pallidum, hippocampus, amygdala, and nucleus accumbens, and ICV were measured also using FreeSurfer. The accuracy of both cortical and subcortical structure segmentation and registration by FreeSurfer was visually checked using the FreeSurfer’s TKMEDIT toolbox.

### Statistical analyses

#### Demographics

Equality of means between the two zygosity groups for the continuous measurements (age, education and mini-mental state examination) was assessed by t-test, and equality of proportion of sexes was assessed by chi-square test. Since the t-test and chi-square test may not be valid in the case of dependent observations (among MZ and DZ pairs), p-values were computed using a permutation procedure (Table 1). Zygosity of pairs was first permutated and then observations from two sets of pairs were interchanged^59^.

#### Heritability

**Figure 1:**
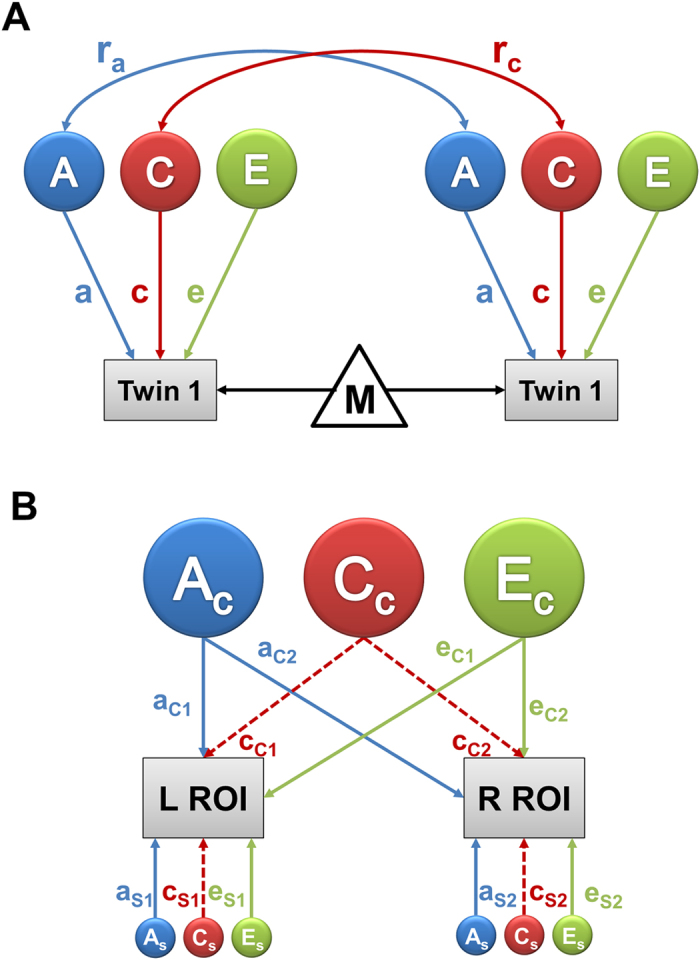
The path diagram for the univariate ACE twin model (**A**) and Independent Pathway Model (IPM) for estimating the overlap between the two hemispheres (L ROI and R ROI) for each of the brain structures (**B**). (**A**) The brain volumetrics of the twin 1 and twin 2 are modelled as the function of the mean parameter (M) and the additive genetic (A), shared environmental (C) and unique environmental (E) factors. The path coefficients a, c and e are the estimated loadings of the latent factors and the variance components corresponding to the factors (E) are respectively denoted as 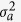, 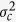.and 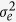. The parameter 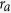 (*r*_*a*_ = 1 for MZ twin pairs and *r*_*a*_ = 0.5 for DZ twin pairs) and *r*_*c*_ (*r*_*a*_ = 1 for both MZ and DZ twin pairs) respectively denote the additive genetic and shared environmental correlation between the twin pairs (**B**). IPM for one of the twins shown here. Additive genetic (A), shared environmental (C), and unique environmental (E) factors; subscript c and s refer respectively to common and specific genetic components of the L (left) and R (right) regions of interest (ROIs). The model is identifiable under the constraint (*a*_*c*1_ = *a*_*c*2_; *c*_*c*1_ = *c*_*c*2_; *e*_c1_ = *e*_*c*2_).

The phenotypic covariance between the twin pairs can be modelled as a function of additive genetic (A), shared environmental (C) and unique environmental (E) components using the mixed effects linear model or SEM. Under SEM, the model containing the three latent factors (A, C and E), known as the ACE model, was fitted to estimate the heritability (Fig. 1A). For parsimony, models containing the components A and E (AE), C and E (CE), and E were compared with the full ACE model using likelihood ratio tests^22^. The path coefficient are denoted (throughout of the manuscript) with lower-case letters *a, c,* and *e* with the corresponding variance components as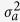, 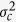, and 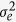. The proportion of variance explained by the additive genetic factor A, (heritability) 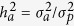, shared environmental factor C, 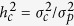 and the unique environmental factor E, 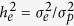; where 
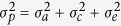
, and the 95% confidence intervals (CI) for the total – left plus right hemisphere – volumes of cortical and subcortical regions, as well as ICV were estimated under the univariate ACE model.

#### Genetic correlation

A bivariate ACE Cholesky model was then used to estimate the genetic correlations (r_G_) between all possible pairs of the seven subcortical and thirty-four cortical regions. Likelihood ratio tests were used to compare the bivariate ACE model with AE, CE and E models. Since the AE model was not parsimonious for all pairs of correlations, the genetic correlations were finally reported based on the ACE model (Supplementary Figure S1).

#### Hemispheric symmetry

In addition to the genetic correlation analysis using a bivariate Cholesky model, an independent pathways model with three common factors (A_c_, C_c_, E_c_) and three specific factors (A_s_, C_s_, E_s_) for each ROI was used to assess the genetic common and specific factors between the corresponding ROIs of the two hemispheres (Fig. 1B). Lateral symmetry of left versus right ROI was tested by constraining the common genetic, shared environmental and unique environmental path coefficients for the two regions to be equal^60^. Under twin designs, large sample sizes are required to estimate the shared environmental components (c_c_ and c_s_)^61^. Hence, to identify the most parsimonious model, the full ACE model was fitted (with a_c1_ = a_c2_; c_c1_ = c_c2_; e_c1_ = e_c2_) and then compared with a reduced model AE (Fig. 1B, constrained with c_c1_ = c_c2_ = 0; c_s1_ = c_s2_ = 0) and CE (a_c1_ = a_c2_ = 0; a_s1_ = a_s2_ = 0) using the likelihood ratio test. For most of the ROIs, the AE model was as good as the ACE model (p-AE > 0.5 and minimum Akaike information cirteria (AIC); Supplementary Table S2); hence, the independent AE model was used to estimate the genetic correlation as well as testing the significance of the genetic correlation r_G_ = 0 and bilateral symmetry (r_G_ = 1).

#### Genetic relationship between cortical lobes and subcortical structures

**Figure 2:**
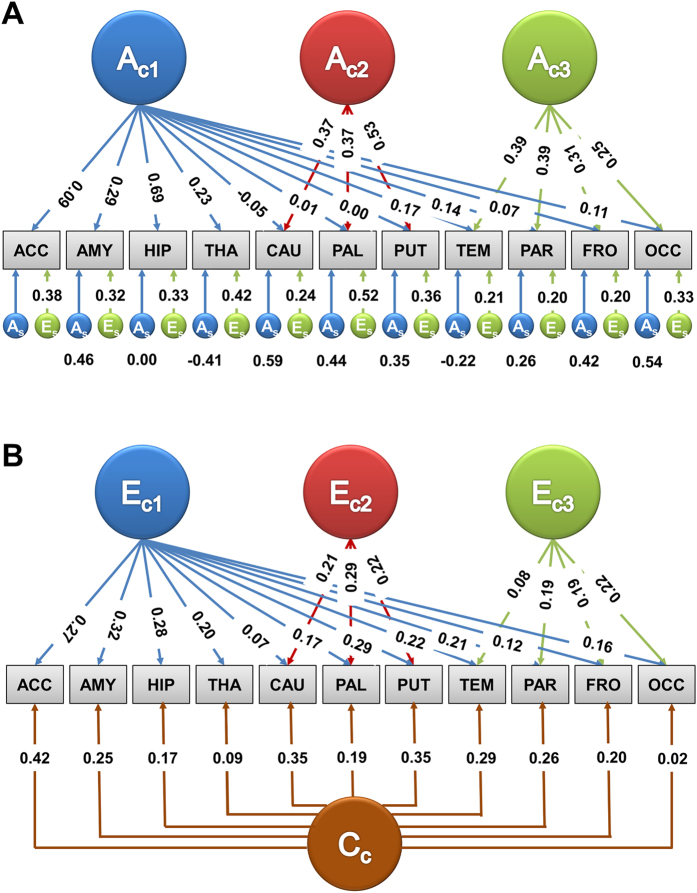
Three factor independent pathway ACE model for correlation between cortical lobar and subcortical volumes. (**A**) Path coefficients for (**A**) common additive genetic factors (top) and specific genetic and environment factors (bottom). (**B**) Shared common environment factor (bottom) and common unique environment factors (top). Note: ACC = Nucleus accumbens; AMY = Amygdala; HIP = Hippocampus; THA = Thalamus; PAL = Pallidum; PAT = Putamen; CAU = Caudate; TEM = Temporal lobe; PAR = Parietal lobe; FRO = Frontal lobe; OCC = Occipital lobe.

**Figure 3:**
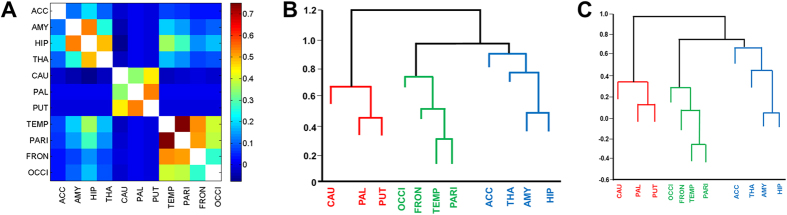
Correlations between cortical lobes and subcortical volumes. (**A**) Genetic correlations between cortical lobes and subcortical volumes using a three factor multivariate IPM. (**B**) Dendrogram constructed from hierarchical clustering of the genetic correlation matrix. (**C**) Dendrogram constructed from hierarchical clustering of the phenotypic correlation matrix. Note: ACC = Nucleus accumbens; AMY = Amygdala; HIP = Hippocampus; THA = Thalamus; PAL = Pallidum; PAT = Putamen; CAU = Caudate; TEM = Temporal lobe; PAR = Parietal lobe; FRO = Frontal lobe; OCC = Occipital lobe.

Our sample size would be too small to employ a full multivariate analysis involving all thirty-four cortical and seven subcortical ROIs. Therefore, for multivariate SEM, we considered only the four major cortical lobes and seven subcortical ROIs. We first obtained the phenotype correlation matrix through the eleven ROI saturated Cholesky model. To construct a parsimonious model with less number of genetic factors we performed hierarchical cluster and factor analysis using the phenotype correlation matrix. We have decided a three component latent factor model for our final solution based on the scree plot of the factors and the hierarchical cluster solution (supplementary Figures S2 and S3). Therefore, we first fitted a three component IPM, and for model parsimony we compared the three factor IPM with some of its sub-models and the ACE Cholesky model. We finally selected a three factor IPM (Fig. 2) with three common additive genetic factors, unique environmental factors, one common shared environmental factor together with ROI specific genetic and environmental components.

The univariate, bivariate and multivariate SEM were carried out using the openMx (2.0.1) R package^62^. Heritability, genetic correlations were estimated after adjusting for the covariates age, sex, scanners and ICV in the means of the univariate and multivariate twins SEM. All the analyses were carried out using the R software^63^.

#### Model selection summary for multivariate SEM model

To evaluate the pattern of genetic clustering among the cortical and subcortical ROIs, we have undertaken a multivariate SEM analysis. Based on the factor and cluster analysis of the phenotype correlation matrix, we decided a three factor IPM for the eleven ROIs. A comparison within the three factor IPMs showed that the model with a single common shared environmental and three genetic and unique environmental factors (IPM2 as shown in Supplementary Table S3) provided a parsimonious fit (minimum AIC and p-value > 0.5) when compared to the other two models.

The IPM was also compared with the ACE Cholesky model. The eleven ROIs AE Cholesky model was as good as the ACE Cholesky model (p-value ≈ 1). Within the Cholesky models, the model with three latent components was not comparable to the eleven latent component model (Supplementary Table S4). However the three factor IPM model (IPM2) was as good as the full Cholesky model and hence the IPM2 model was selected as the final model for our analysis.

## Additional Information

**How to cite this article**: Wen, W. *et al.* Distinct Genetic Influences on Cortical and Subcortical Brain Structures. *Sci. Rep.* **6**, 32760; doi: 10.1038/srep32760 (2016).

## Supplementary Material

Supplementary Information

## Figures and Tables

**Table 1:** Demographics of the sample.

	MZ (n = 186)	DZ (n = 136)	Total (n = 322)	Statistic	P*
Sex Females (%)	120 (64.5%)	95 (69.9%)	215 (66.8)	0.780#	0.157
Age, mean (SD)	70.2 (5.1)	69.9 (4.6)	70.1 (4.9)	0.484¶	0.620
Education (y) mean (SD)	11.0 (3.3)	11.1 (3.3)	11.0 (3.3)	0.206¶	0.833
MMSE mean (SD)	28.5 (2.0)	28.8 (1.3)	28.6 (1.7)	1.698¶	0.091

Note: Mini-Mental State Examination (MMSE) (Folstein *et al.*^23^); monozygotic (MZ); dizygotic (DZ).

^#^Chi-squared test.

^¶^T-test.

^*^All p-values were obtained by 10000 permutations.

**Table 2:** Twin pair correlations (95% confidence intervals) and heritability estimates for total volumes of four cortical lobar regions, seven subcortical structures and intracranial volume (ICV).

Region	Within twin pair intraclass correlations (95% CI)		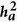	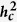	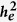	Covariates Significance
	MZ pairs (N = 93)	DZ pairs (N = 68)				
Cortical Lobar Regions						
Frontal cortex	0.79 (0.70,0.85)	0.40 (0.35,0.57)	0.78 (0.42,0.85)	0.01 (0,0.35)	0.21 (0.15,0.3)	sNSS
Temporal cortex	0.75 (0.65,0.82)	0.46 (0.33,0.62)	0.58 (0.24,0.81)	0.17 (0,0.49)	0.25 (0.18,0.35)	sNSS
Parietal cortex	0.70 (0.58,0.78)	0.42 (0.30,0.59)	0.55 (0.18,0.78)	0.15 (0,0.48)	0.30 (0.22,0.42)	sNSS
Occipital cortex	0.70 (0.59,0.78)	0.35 (0.29,0.48)	0.70 (0.42,0.78)	0.00 (0,0.25)	0.30 (0.22,0.41)	sNSS
Subcortical structures						
Nucleus accumbens	0.65 (0.53,0.74)	0.44 (0.28,0.61)	0.42 (0.03,0.73)	0.24 (0,0.57)	0.35 (0.26,0.47)	sNSN
Amygdala	0.61 (0.48,0.71)	0.41 (0.26,0.58)	0.41 (0.00,0.71)	0.21 (0,0.56)	0.39 (0.29,0.52)	sNSS
Caudate	0.86 (0.79,0.90)	0.52 (0.40,0.66)	0.67 (0.39,0.89)	0.19 (0,0.46)	0.14 (0.1,0.21)	NsSS
Hippocampus	0.73 (0.63,0.81)	0.37 (0.31,0.47)	0.73 (0.52,0.81)	0.00 (0,0.19)	0.27 (0.19,0.37)	sNSS
Pallidum	0.49 (0.33,0.62)	0.24 (0.17,0.40)	0.49 (0.11,0.62)	0.00 (0,0.32)	0.51 (0.38,0.67)	sSNS
Putamen	0.68 (0.55,0.77)	0.34 (0.28,0.5)	0.68 (0.32,0.77)	0.00 (0,0.32)	0.32 (0.23,0.45)	sNSS
Thalamus	0.50 (0.35,0.63)	0.25 (0.18,0.44)	0.51 (0.07,0.63)	0.00 (0,0.38)	0.50 (0.37,0.65)	sNSS
ICV	0.88 (0.83,0.91)	0.48 (0.41,0.63)	0.79 (0.50,0.91)	0.09 (0,0.37)	0.12 (0.09,0.17)	NSS*

Univariate ACE model intra-class correlation (ICC) with 95% CI and heritability (95% CI) for total volumes (left plus right hemisphere) of four cortical lobar regions, seven subcortical structures and intracranial volume (ICV). All estimates were adjusted for different scanners, age, sex and ICV where appropriate. Last column indicates the significance of covariates. Significance of the p-value (p < 0.05) for any of the covariates age, sex (coded 1 for male and 0 for female), scanners (4 scanners coded with 3 dummy variables) and ICV in that order is indicated as a string; S = significant and beta >0; s =  significant and beta <0; N = not significant and * = not applicable. Significance for scanners were coded as S if p < 0.05 for any one of the scanners, ignoring the direction of beta.

**Table 3:** Genetic (upper right triangle) and environmental (lower triangle) correlations between cortical lobes and subcortical structures.

	ACC	AMY	HIP	THA	CAU	PAL	PUT	TEMP	PARI	FRON	OCCI
ACC		*0.11*	*0.21*	*0.10*	−*0.02*	*0.00*	*0.00*	0.07	0.06	0.03	0.04
AMY	*0.41*		*0.54*	*0.26*	−*0.04*	*0.01*	*0.00*	0.19	0.15	0.07	0.10
HIP	*0.38*	*0.46*		*0.49*	−*0.08*	*0.01*	*0.00*	0.36	0.28	0.14	0.18
THA	*0.24*	*0.29*	*0.27*		*−0.04*	*0.01*	*0.00*	0.17	0.14	0.07	0.09
CAU	*0.12*	*0.15*	*0.14*	*0.09*		*0.35*	*0.44*	0.03	−0.02	−0.01	−0.01
PAL	*0.16*	*0.19*	*0.18*	*0.12*	*0.36*		*0.54*	0.00	0.00	0.00	0.00
PUT	*0.33*	*0.39*	*0.36*	*0.24*	*0.40*	*0.36*		0.00	0.00	0.00	0.00
TEMP	0.40	0.49	0.45	0.29	0.15	0.19	0.39		**0.75**	**0.53**	**0.40**
PARI	0.35	0.42	0.39	0.25	0.12	0.16	0.33	**0.56**		**0.51**	**0.38**
FRON	0.23	0.28	0.26	0.16	0.08	0.11	0.22	**0.45**	**0.59**		**0.27**
OCCI	0.21	0.26	0.24	0.15	0.08	0.10	0.21	**0.39**	**0.50**	**0.48**	

Note: ACC = Nucleus accumbens; AMY = Amygdala; HIP = Hippocampus; THA = Thalamus; CAU = Caudate; PAL = Pallidum; PAT = Putamen; FRON = Frontal lobe; TEMP = Temporal lobe; PARI = Parietal lobe; OCCI = Occipital lobe. The genetic correlation coefficients are graphically presented in Fig. 3A in the form of heat map. Numbers in bold, italics and plain text highlights the correlations for the cortical lobes, subcortical structures and between the subcortical structures and cortical lobes respectively.
