# Supplementary material for: Distinct Genetic Influences on Cortical and Subcortical Brain Structures
Source: Sci Rep. 2016 Sep 6;6:32760. doi: 10.1038/srep32760 (PMC5011703; doi:10.1038/srep32760)
Supplement: Supplementary Information [file srep32760-s1.pdf]

SUPPLEMENTARY INFORMATION

DISTINCT GENETIC INFLUENCES ON CORTICAL AND SUBCORTICAL BRAIN STRUCTURES

Wei Wen[1,2,#], Anbupalam Thalamuthu[1,#], Karen A. Mather[1], Wanlin Zhu[1], Jiyang Jiang[1], Pierre Lafaye de Micheaux[1,3], Margaret J. Wright[4,5], David Ames[6], Perminder S. Sachdev[1,2,*]

[1]*Centre for Healthy Brain Ageing, School of Psychiatry, University of New South Wales,*

Sydney, NSW 2052, Australia

[2]*Neuropsychiatric Institute, Prince of Wales Hospital, Randwick, NSW 2031, Australia*

[3]*Department of Mathematics and Statistics, University of Montreal, Montreal, QC H3T1J4, Canada*

[4] *Queensland Brain Institute, University of Queensland, St Lucia, QLD 4072, Australia*

[5] *Neuroimaging Genetics, QIMR Berghofer Medical Research Institute, Brisbane, QLD 4006, Australia*

[6] *National Aging Research Institute, University of Melbourne, Parkville, VIC 3052, Australia*

[#]*These two authors equally contributed to the work.*

Supplementary Table S1. Twin pair correlations (95% confidence intervals) and heritability estimates for all cortical ROIs.
Univariate ACE model intra-class correlation (ICC) with 95% CI and heritability (95% CI) for four cortical lobar regions, seven subcortical structures and intracranial volume (ICV). All estimates were adjusted for different scanners, age, sex and ICV where appropriate. Last column indicates the significance of covariates. Significance of the p-value (p<0.05) for any of the covariates age, sex (coded 1 for male and 0 for female), scanners (4 scanners coded with 3 dummy variables) and ICV in that order is indicated as a string; S=significant and beta >0; s= significant and beta <0; N=not-significant. (**A**) For total volumes obtained as the sum of the same structures on ***both*** hemispheres. (**B**) For the volumes of ***left*** hemisphere. (**C**) For the volumes of ***right*** hemisphere. Significance for scanners were coded as S if p<0.05 for any one of the scanners, ignoring the direction of beta.

A

Region of Interest (ROI)		Within twin pair intraclass correlations (95% CI)		h_a^2	h_c^2	h_e^2	Covariates Significance
		MZ pairs (N= 93)	DZ pairs (N=68)				
Frontal Lobe ROIs	Caudal anterior-cingulate cortex	0.43 (0.29,0.55)	0.43 (0.20,0.55)	0.00 (0.00,0.47)	0.43 (0.00,0.55)	0.57 (0.45,0.71)	sNNS
	Caudal middle frontal gyrus	0.58 (0.44,0.69)	0.40 (0.24,0.58)	0.35 (0.00,0.68)	0.22 (0.00,0.58)	0.42 (0.31,0.56)	sNSS
	Frontal pole	0.40 (0.22,0.54)	0.20 (0.11,0.37)	0.40 (0.00,0.54)	0.00 (0.00,0.35)	0.60 (0.46,0.78)	NNSS
	Lateral orbital frontal cortex	0.56 (0.41,0.67)	0.33 (0.21,0.51)	0.47 (0.01,0.67)	0.09 (0.00,0.48)	0.44 (0.33,0.59)	sNSS
	Medial orbital frontal cortex	0.46 (0.29,0.60)	0.23 (0.14,0.38)	0.46 (0.07,0.60)	0.00 (0.00,0.30)	0.54 (0.40,0.71)	NNSS
	Paracentral lobule	0.69 (0.58,0.77)	0.37 (0.29,0.55)	0.65 (0.24,0.77)	0.04 (0.00,0.41)	0.31 (0.23,0.42)	NsSS
	Pars opercularis	0.47 (0.30,0.60)	0.23 (0.15,0.36)	0.47 (0.16,0.60)	0.00 (0.00,0.24)	0.53 (0.40,0.70)	ssSS
	Pars orbitalis	0.50 (0.34,0.63)	0.25 (0.17,0.36)	0.50 (0.23,0.63)	0.00 (0.00,0.21)	0.50 (0.37,0.66)	NNSS
	Pars triangularis	0.43 (0.26,0.57)	0.22 (0.13,0.39)	0.43 (0.00,0.57)	0.00 (0.00,0.37)	0.57 (0.43,0.74)	NNSS
	Precentral gyrus	0.67 (0.55,0.76)	0.34 (0.28,0.52)	0.67 (0.27,0.76)	0.00 (0.00,0.36)	0.33 (0.24,0.45)	sNSS
	Rostral anterior cingulate cortex	0.58 (0.44,0.68)	0.29 (0.22,0.50)	0.58 (0.11,0.68)	0.00 (0.00,0.00)	0.42 (0.32,0.56)	sNSS
	Rostral middle frontal gyrus	0.60 (0.46,0.70)	0.30 (0.23,0.45)	0.60 (0.23,0.70)	0.00 (0.00,0.31)	0.40 (0.30,0.54)	NNSS
	Superior frontal gyrus	0.59 (0.45,0.70)	0.30 (0.22,0.43)	0.59 (0.28,0.70)	0.00 (0.00,0.26)	0.41 (0.30,0.55)	NsNS
Parietal Lobe ROIs	Inferior parietal cortex	0.50 (0.35,0.63)	0.25 (0.17,0.43)	0.50 (0.08,0.63)	0.00 (0.00,0.36)	0.50 (0.37,0.65)	sNNS
	Isthmus - cingulate cortex	0.52 (0.36,0.65)	0.32 (0.20,0.50)	0.40 (0.00,0.65)	0.12 (0.00,0.49)	0.48 (0.35,0.64)	sNSS
	Postcentral gyrus	0.63 (0.51,0.73)	0.32 (0.25,0.50)	0.63 (0.22,0.73)	0.00 (0.00,0.37)	0.37 (0.27,0.49)	sNSS
	Posterior-cingulate cortex	0.66 (0.52,0.76)	0.33 (0.26,0.44)	0.66 (0.41,0.76)	0.00 (0.00,0.21)	0.34 (0.24,0.48)	sNSS

	Precuneus cortex	0.59 (0.45,0.69)	0.34 (0.23,0.53)	0.50 (0.06,0.69)	0.08 (0.00,0.47)	0.41 (0.31,0.55)	sNSS
	Superior parietal cortex	0.62 (0.49,0.73)	0.31 (0.24,0.42)	0.62 (0.38,0.73)	0.00 (0.00,0.00)	0.38 (0.27,0.51)	sNSS
	Supramarginal gyrus	0.64 (0.51,0.73)	0.32 (0.26,0.51)	0.64 (0.20,0.73)	0.00 (0.00,0.39)	0.36 (0.27,0.49)	sNSS
Temporal Lobe ROIs	Banks superior temporal sulcus	0.48 (0.31,0.61)	0.27 (0.16,0.47)	0.41 (0.00,0.61)	0.07 (0.00,0.47)	0.52 (0.39,0.69)	sNSS
	Entorhinal cortex	0.30 (0.13,0.44)	0.15 (0.07,0.39)	0.30 (0.00,0.44)	0.00 (0.00,0.39)	0.70 (0.56,0.87)	sNSS
	Fusiform gyrus	0.67 (0.54,0.76)	0.34 (0.27,0.52)	0.66 (0.25,0.76)	0.01 (0.00,0.37)	0.33 (0.24,0.46)	sNSS
	Inferior temporal gyrus	0.58 (0.44,0.69)	0.29 (0.22,0.49)	0.58 (0.13,0.69)	0.00 (0.00,0.39)	0.42 (0.31,0.56)	sNSS
	Middle temporal gyrus	0.65 (0.52,0.75)	0.33 (0.26,0.42)	0.65 (0.43,0.75)	0.00 (0.00,0.18)	0.35 (0.25,0.48)	sNSS
	Parahippocampal gyrus	0.66 (0.52,0.76)	0.33 (0.26,0.42)	0.66 (0.44,0.76)	0.00 (0.00,0.18)	0.34 (0.24,0.48)	sNNS
	Superior temporal gyrus	0.64 (0.50,0.74)	0.32 (0.25,0.42)	0.64 (0.41,0.74)	0.00 (0.00,0.19)	0.36 (0.26,0.50)	NNSS
	Temporal pole	0.41 (0.27,0.56)	0.39 (0.18,0.53)	0.04 (0.00,0.53)	0.37 (0.00,0.53)	0.59 (0.44,0.73)	sNSS
	Transverse temporal cortex	0.52 (0.38,0.64)	0.47 (0.24,0.61)	0.10 (0.00,0.59)	0.41 (0.00,0.61)	0.48 (0.36,0.62)	sNSS
Occipital Lobe ROIs	Cuneus cortex	0.63 (0.49,0.73)	0.37 (0.26,0.55)	0.52 (0.10,0.73)	0.11 (0.00,0.47)	0.37 (0.27,0.51)	sNSS
	Lateral occipital cortex	0.54 (0.40,0.66)	0.27 (0.20,0.44)	0.54 (0.14,0.66)	0.00 (0.00,0.35)	0.46 (0.34,0.60)	sNSS
	Lingual gyrus	0.64 (0.50,0.74)	0.32 (0.25,0.43)	0.64 (0.38,0.74)	0.00 (0.00,0.21)	0.36 (0.26,0.50)	sNSS
	Pericalcarine cortex	0.64 (0.51,0.73)	0.45 (0.28,0.61)	0.38 (0.00,0.72)	0.26 (0.00,0.59)	0.36 (0.27,0.49)	NNSS
Insular Cortex	Insula cortex	0.67 (0.54,0.76)	0.33 (0.27,0.45)	0.67 (0.40,0.76)	0.00 (0.00,0.23)	0.33 (0.24,0.46)	sNNS

B

Region of Interest (ROI)		Within twin pair intraclass correlations (95% CI)		h_a^2	h_c^2	h_e^2	Covariates Significance
		MZ pairs (N= 93)	DZ pairs (N=68)				
Frontal Lobe ROIs	Caudal anterior-cingulate cortex	0.15 (0.00,0.31)	0.08 (0.00,0.28)	0.14 (0.00,0.31)	0.01 (0.00,0.28)	0.85 (0.69,1.00)	sNNS
	Caudal middle frontal gyrus	0.45 (0.31,0.58)	0.45 (0.20,0.56)	0.00 (0.00,0.53)	0.45 (0.00,0.56)	0.55 (0.42,0.69)	sNSS
	Frontal pole	0.24 (0.05,0.41)	0.12 (0.03,0.31)	0.24 (0.00,0.41)	0.00 (0.00,0.31)	0.76 (0.59,0.95)	NNSS
	Lateral orbital frontal cortex	0.55 (0.40,0.67)	0.28 (0.20,0.43)	0.55 (0.16,0.67)	0.00 (0.00,0.32)	0.45 (0.33,0.60)	sNSS
	Medial orbital frontal cortex	0.31 (0.11,0.47)	0.15 (0.06,0.35)	0.31 (0.00,0.47)	0.00 (0.00,0.35)	0.69 (0.53,0.89)	NNSS
	Paracentral lobule	0.53 (0.37,0.65)	0.27 (0.19,0.44)	0.53 (0.10,0.65)	0.00 (0.00,0.35)	0.47 (0.35,0.63)	NsSS
	Pars opercularis	0.37 (0.19,0.52)	0.19 (0.10,0.36)	0.37 (0.00,0.52)	0.00 (0.00,0.35)	0.63 (0.48,0.81)	sNSS
	Pars orbitalis	0.28 (0.08,0.46)	0.14 (0.04,0.28)	0.28 (0.00,0.46)	0.00 (0.00,0.28)	0.72 (0.54,0.92)	sNSS
	Pars triangularis	0.22 (0.03,0.40)	0.11 (0.01,0.26)	0.22 (0.00,0.40)	0.00 (0.00,0.26)	0.78 (0.60,0.97)	NNSS
	Precentral gyrus	0.61 (0.47,0.71)	0.30 (0.24,0.42)	0.61 (0.34,0.71)	0.00 (0.00,0.23)	0.39 (0.29,0.53)	sNSS
	Rostral anterior cingulate cortex	0.44 (0.28,0.57)	0.22 (0.14,0.46)	0.44 (0.00,0.57)	0.00 (0.00,0.46)	0.56 (0.43,0.72)	sNNS
	Rostral middle frontal gyrus	0.58 (0.43,0.69)	0.29 (0.22,0.44)	0.58 (0.23,0.69)	0.00 (0.00,0.30)	0.42 (0.31,0.57)	NNSS
	Superior frontal gyrus	0.61 (0.47,0.72)	0.31 (0.24,0.43)	0.61 (0.33,0.72)	0.00 (0.00,0.24)	0.39 (0.28,0.53)	NNSS
Parietal ROIs	Inferior parietal cortex	0.42 (0.25,0.57)	0.21 (0.13,0.32)	0.42 (0.15,0.57)	0.00 (0.00,0.21)	0.58 (0.43,0.75)	sNNS
	Isthmus - cingulate cortex	0.37 (0.19,0.53)	0.25 (0.11,0.44)	0.23 (0.00,0.53)	0.14 (0.00,0.44)	0.63 (0.47,0.81)	NNSS
	Postcentral gyrus	0.49 (0.33,0.62)	0.25 (0.17,0.42)	0.49 (0.08,0.62)	0.00 (0.00,0.35)	0.51 (0.38,0.67)	NNSS
	Posterior-cingulate cortex	0.37 (0.17,0.53)	0.18 (0.09,0.34)	0.37 (0.00,0.53)	0.00 (0.00,0.32)	0.63 (0.47,0.83)	sNSS
	Precuneus cortex	0.45 (0.28,0.59)	0.32 (0.16,0.51)	0.25 (0.00,0.58)	0.20 (0.00,0.51)	0.55 (0.41,0.72)	sNSS
	Superior parietal cortex	0.46 (0.30,0.59)	0.23 (0.15,0.39)	0.46 (0.08,0.59)	0.00 (0.00,0.31)	0.54 (0.41,0.70)	sNSS
	Supramarginal gyrus	0.50 (0.34,0.62)	0.32 (0.18,0.52)	0.35 (0.00,0.62)	0.15 (0.00,0.52)	0.50 (0.38,0.66)	sNSS
Temporal ROIs	Banks superior temporal sulcus	0.35 (0.17,0.51)	0.22 (0.10,0.42)	0.27 (0.00,0.51)	0.08 (0.00,0.42)	0.65 (0.49,0.83)	sNNS
	Entorhinal cortex	0.29 (0.12,0.44)	0.15 (0.06,0.38)	0.29 (0.00,0.44)	0.00 (0.00,0.38)	0.71 (0.56,0.88)	NNSS
	Fusiform gyrus	0.56 (0.40,0.68)	0.28 (0.20,0.37)	0.56 (0.33,0.68)	0.00 (0.00,0.17)	0.44 (0.32,0.60)	sNSS
	Inferior temporal gyrus	0.47 (0.30,0.60)	0.23 (0.15,0.38)	0.47 (0.10,0.60)	0.00 (0.00,0.30)	0.53 (0.40,0.70)	NNSS
	Middle temporal gyrus	0.45 (0.27,0.59)	0.22 (0.14,0.34)	0.45 (0.15,0.59)	0.00 (0.00,0.22)	0.55 (0.41,0.73)	sNSS
	Parahippocampal gyrus	0.67 (0.55,0.76)	0.34 (0.27,0.46)	0.67 (0.39,0.76)	0.00 (0.00,0.24)	0.33 (0.24,0.45)	sNSS

	Superior temporal gyrus	0.57 (0.41,0.69)	0.28 (0.21,0.37)	0.57 (0.37,0.69)	0.00 (0.00,0.15)	0.43 (0.31,0.59)	sNSS
	Temporal pole	0.34 (0.19,0.50)	0.31 (0.12,0.46)	0.07 (0.00,0.49)	0.27 (0.00,0.46)	0.66 (0.50,0.81)	NNSN
	Transverse temporal cortex	0.47 (0.30,0.60)	0.26 (0.16,0.46)	0.41 (0.00,0.60)	0.06 (0.00,0.46)	0.53 (0.40,0.70)	sNNS
Occipital	Cuneus cortex	0.54 (0.40,0.66)	0.36 (0.21,0.56)	0.36 (0.00,0.65)	0.18 (0.00,0.56)	0.46 (0.34,0.60)	sNSS
	Lateral occipital cortex	0.46 (0.28,0.59)	0.24 (0.15,0.44)	0.44 (0.00,0.59)	0.02 (0.00,0.44)	0.54 (0.41,0.72)	NNSS
	Lingual gyrus	0.54 (0.38,0.67)	0.27 (0.19,0.37)	0.54 (0.30,0.67)	0.00 (0.00,0.18)	0.46 (0.33,0.62)	sNSS
	Pericalcarine cortex	0.58 (0.44,0.69)	0.33 (0.23,0.53)	0.49 (0.03,0.69)	0.09 (0.00,0.49)	0.42 (0.31,0.56)	NNSS
Insular	Insula cortex	0.64 (0.50,0.74)	0.32 (0.25,0.43)	0.64 (0.37,0.74)	0.00 (0.00,0.22)	0.36 (0.26,0.50)	NNSS

C

**Region of Interest (ROI)**		**Within twin pair intraclass correlations (95% CI)**		h_a^2	h_c^2	h_e^2	**Covariates Significance**
		MZ pairs (N= 93)	DZ pairs (N=68)				
Frontal Lobe ROIs	Caudal anterior-cingulate cortex	0.21 (0.04,0.38)	0.18 (0.02,0.34)	0.06 (0.00,0.38)	0.14 (0.00,0.34)	0.79 (0.62,0.96)	sNNS
	Caudal middle frontal gyrus	0.35 (0.16,0.50)	0.20 (0.09,0.41)	0.29 (0.00,0.50)	0.06 (0.00,0.41)	0.65 (0.50,0.84)	NsSS
	Frontal pole	0.31 (0.11,0.47)	0.16 (0.06,0.35)	0.31 (0.00,0.47)	0.00 (0.00,0.35)	0.69 (0.53,0.89)	NNSN
	Lateral orbital frontal cortex	0.42 (0.26,0.56)	0.32 (0.15,0.50)	0.21 (0.00,0.56)	0.21 (0.00,0.50)	0.58 (0.44,0.74)	NNSS
	Medial orbital frontal cortex	0.33 (0.14,0.49)	0.16 (0.07,0.28)	0.33 (0.02,0.49)	0.00 (0.00,0.22)	0.67 (0.51,0.86)	NNSS
	Paracentral lobule	0.59 (0.46,0.70)	0.37 (0.24,0.56)	0.45 (0.01,0.70)	0.14 (0.00,0.53)	0.41 (0.30,0.54)	NNSS
	Pars opercularis	0.31 (0.13,0.46)	0.15 (0.06,0.31)	0.31 (0.00,0.46)	0.00 (0.00,0.31)	0.69 (0.54,0.87)	NNSS
	Pars orbitalis	0.37 (0.20,0.52)	0.18 (0.10,0.30)	0.37 (0.10,0.52)	0.00 (0.00,0.21)	0.63 (0.48,0.80)	NNNS
	Pars triangularis	0.38 (0.21,0.53)	0.19 (0.10,0.35)	0.38 (0.00,0.53)	0.00 (0.00,0.33)	0.62 (0.47,0.79)	sNSS
	Precentral gyrus	0.50 (0.35,0.62)	0.34 (0.19,0.54)	0.31 (0.00,0.62)	0.19 (0.00,0.54)	0.50 (0.38,0.65)	sNSS
	Rostral anterior cingulate cortex	0.42 (0.25,0.55)	0.21 (0.13,0.40)	0.42 (0.00,0.55)	0.00 (0.00,0.40)	0.58 (0.45,0.75)	NNSS
	Rostral middle frontal gyrus	0.44 (0.27,0.58)	0.22 (0.14,0.41)	0.44 (0.00,0.58)	0.00 (0.00,0.40)	0.56 (0.42,0.73)	NNSS
	Superior frontal gyrus	0.46 (0.30,0.60)	0.23 (0.15,0.36)	0.46 (0.13,0.60)	0.00 (0.00,0.27)	0.54 (0.40,0.70)	NNSS
Parietal ROIs	Inferior parietal cortex	0.38 (0.20,0.53)	0.25 (0.12,0.44)	0.27 (0.00,0.53)	0.11 (0.00,0.44)	0.62 (0.47,0.80)	sNNS
	Isthmus - cingulate cortex	0.44 (0.27,0.58)	0.22 (0.14,0.43)	0.44 (0.00,0.58)	0.00 (0.00,0.43)	0.56 (0.42,0.73)	NNSS
	Postcentral gyrus	0.49 (0.33,0.62)	0.26 (0.17,0.46)	0.47 (0.00,0.62)	0.02 (0.00,0.45)	0.51 (0.38,0.67)	sNNS

	Posterior-cingulate cortex	0.49 (0.32,0.62)	0.24 (0.16,0.41)	0.49 (0.06,0.62)	0.00 (0.00,0.35)	0.51 (0.38,0.68)	sNSS
	Precuneus cortex	0.53 (0.38,0.65)	0.29 (0.20,0.50)	0.48 (0.00,0.65)	0.06 (0.00,0.48)	0.47 (0.35,0.62)	sNSS
	Superior parietal cortex	0.40 (0.23,0.54)	0.20 (0.12,0.37)	0.40 (0.00,0.54)	0.00 (0.00,0.34)	0.60 (0.46,0.77)	sNSS
	Supramarginal gyrus	0.53 (0.38,0.65)	0.27 (0.19,0.43)	0.53 (0.15,0.65)	0.00 (0.00,0.33)	0.47 (0.35,0.62)	sNSS
Temporal ROIs	Banks superior temporal sulcus	0.15 (0.00,0.33)	0.11 (0.00,0.29)	0.07 (0.00,0.33)	0.08 (0.00,0.29)	0.85 (0.67,1.00)	sNNS
	Entorhinal cortex	0.21 (0.03,0.37)	0.10 (0.02,0.28)	0.21 (0.00,0.37)	0.00 (0.00,0.28)	0.79 (0.63,0.97)	NNNS
	Fusiform gyrus	0.49 (0.33,0.63)	0.41 (0.22,0.56)	0.17 (0.00,0.60)	0.32 (0.00,0.56)	0.51 (0.37,0.67)	sNSS
	Inferior temporal gyrus	0.40 (0.22,0.54)	0.28 (0.13,0.47)	0.23 (0.00,0.54)	0.17 (0.00,0.47)	0.60 (0.46,0.78)	sNSS
	Middle temporal gyrus	0.58 (0.43,0.70)	0.29 (0.22,0.41)	0.58 (0.30,0.70)	0.00 (0.00,0.23)	0.42 (0.30,0.57)	sNSS
	Parahippocampal gyrus	0.41 (0.24,0.56)	0.21 (0.12,0.32)	0.41 (0.12,0.56)	0.00 (0.00,0.22)	0.59 (0.44,0.76)	sNSS
	Superior temporal gyrus	0.47 (0.31,0.60)	0.23 (0.16,0.42)	0.47 (0.02,0.60)	0.00 (0.00,0.38)	0.53 (0.40,0.69)	ssSS
	Temporal pole	0.27 (0.12,0.43)	0.27 (0.08,0.40)	0.00 (0.00,0.42)	0.27 (0.00,0.40)	0.73 (0.57,0.88)	NNNS
	Transverse temporal cortex	0.27 (0.12,0.43)	0.27 (0.07,0.41)	0.00 (0.00,0.42)	0.27 (0.00,0.41)	0.73 (0.57,0.88)	sNNS
Occipital	Cuneus cortex	0.54 (0.36,0.67)	0.30 (0.19,0.47)	0.48 (0.01,0.67)	0.06 (0.00,0.43)	0.46 (0.33,0.64)	sNSS
	Lateral occipital cortex	0.42 (0.25,0.56)	0.21 (0.12,0.34)	0.42 (0.09,0.56)	0.00 (0.00,0.27)	0.58 (0.44,0.75)	sNSS
	Lingual gyrus	0.48 (0.30,0.61)	0.24 (0.16,0.42)	0.48 (0.00,0.61)	0.00 (0.00,0.38)	0.52 (0.39,0.70)	sNSS
	Pericalcarine cortex	0.54 (0.38,0.66)	0.43 (0.24,0.58)	0.22 (0.00,0.64)	0.32 (0.00,0.58)	0.46 (0.34,0.62)	NSSS
Insular	Insula cortex	0.56 (0.40,0.68)	0.28 (0.20,0.39)	0.56 (0.29,0.68)	0.00 (0.00,0.21)	0.44 (0.32,0.60)	NNSS

Supplementary Table S2.

Bilateral symmetry of ROIs of the two hemispheres (left=H & right=H)

The results in this table were obtained based on the bivariate AE IPM described in Figure 1B. Summarized are the unstandardized variance components (square of path-coefficients) corresponding to common (subscript c) and specific (subscript s) genetic and environment components obtained under the constrained model for bilateral symmetry (ac1=ac2; ec1=ec2). The genetic rG (95% confidence interval) and environmental rE (95% confidence interval) correlations were obtained under the AE model. The columns p-AE, p-CE and p-E respectively denote the p-values from the likelihood ratio test comparing ACE model vs AE, CE and E models. p-AE_com is the p-value obtained from the likelihood ratio tests for the comparison of the AE model with the constrained model (as1 = as2=0). The significance of the genetic sharing was assessed through the likelihood ratio test of the AE model with the common paths (ac1 = ac2) vs the reduced model (ac1 = ac2 = 0) model (p-AE_as). Test of hypothesis r_G=1 was assessed through the likelihood ratio test of the AE model vs the AE model with the additional constraint as1 = as1 (p-AE_asc). The p-values were rounded to 2 decimal places and hence 0.00 denotes p<10-2. *CE model was better than AE model (Three instances: Caudal anterior-cingulate cortex, Temporal pole and Transverse temporal cortex). #supported by minimum AIC criteria for model comparison.

**Region of Interest**	$\sigma^2_{a_c}$	$\sigma^2_{e_c}$	**L** $\sigma^2_{a_s}$	**R** $\sigma^2_{a_s}$	**L** $\sigma^2_{e_s}$	**R** $\sigma^2_{e_s}$	**r_G (95% CI)**	**r_E(95% CI)**	**p-AE**	**p-CE**	**p-E**	**p-AE_com**	**p-AE_as**	**p-AE_asc**
**Cortical ROIs**														
Banks superior temporal sulcus	0.19	0.00	0.09	0.00	0.50	0.57	0.82 (0.55,1.00)	0.00 (0.00,0.14)	0.99	0.76	0.00	0.41	0.00	0.18
Caudal anterior-cingulate cortex	0.12	0.00	0.00	0.06	0.78	0.72	0.83 (0.25,1.00)	0.00 (0.00,0.05)	0.60	1.00*#	0.00	0.79	0.01	1.00
Caudal middle frontal gyrus	0.32	0.04	0.00	0.00	0.31	0.40	1.00 (0.88,1.00)	0.11 (0.00,0.26)	0.92	0.62	0.00	1.00	0.00	1.00
Cuneus cortex	0.31	0.08	0.08	0.11	0.22	0.27	0.77 (0.61,0.92)	0.24 (0.06,0.41)	0.98	0.18	0.00	0.01#	0.00	0.09#
Entorhinal cortex	0.13	0.07	0.05	0.03	0.38	0.59	0.77 (0.29,1.00)	0.13 (0.00,0.29)	1.00	0.81	0.03	0.64	0.02	0.38
Fusiform gyrus	0.38	0.05	0.00	0.00	0.27	0.29	1.00 (0.89,1.00)	0.14 (0.00,0.30)	1.00	0.08	0.00	1.00	0.00	1.00
Inferior parietal cortex	0.25	0.10	0.02	0.01	0.27	0.29	0.95 (0.74,1.00)	0.26 (0.09,0.42)	1.00	0.19	0.00	0.86	0.00	0.67
Inferior temporal gyrus	0.25	0.04	0.00	0.00	0.26	0.32	0.99 (0.78,1.00)	0.12 (0.00,0.29)	1.00	0.17	0.00	1.00	0.00	0.97
Isthmus - cingulate cortex	0.24	0.08	0.03	0.11	0.33	0.34	0.78 (0.54,0.98)	0.19 (0.01,0.36)	0.98	0.39	0.00	0.11#	0.00	0.58
Lateral	0.27	0.09	0.02	0.00	0.27	0.26	0.96 (0.791.00)	0.26 (0.10,0.41)	1.00	0.15	0.00	0.90	0.00	0.64

occipital cortex														
Lateral orbital frontal cortex	0.24	0.07	0.02	0.00	0.18	0.18	0.96 (0.84,1.00)	0.29 (0.13,0.44)	1.00	0.25	0.00	0.84	0.00	0.56
Lingual gyrus	0.38	0.07	0.05	0.00	0.29	0.31	0.94 (0.79,1.00)	0.19 (0.02,0.36)	1.00	0.01	0.00	0.60	0.00	0.31
Medial orbital frontal cortex	0.17	0.04	0.00	0.02	0.28	0.34	0.96 (0.68,1.00)	0.12 (0.00,0.29)	1.00	0.20	0.00	0.95	0.00	1.00
Middle temporal gyrus	0.30	0.07	0.01	0.04	0.28	0.16	0.93 (0.79,1.00)	0.24 (0.06,0.41)	1.00	0.00	0.00	0.52	0.00	0.86
Parahippocam pal gyrus	0.35	0.06	0.19	0.02	0.20	0.43	0.78 (0.60,0.91)	0.17 (0.00,0.35)	1.00	0.00	0.00	0.00#	0.00	0.00#
Paracentral lobule	0.39	0.01	0.00	0.06	0.34	0.28	0.93 (0.79,1.00)	0.03 (0.00,0.21)	0.99	0.02	0.00	0.49	0.00	1.00
Pars opercularis	0.26	0.08	0.01	0.00	0.36	0.46	0.98 (0.70,1.00)	0.16 (0.00,0.33)	1.00	0.12	0.00	0.98	0.00	0.85
Pars orbitalis	0.30	0.08	0.00	0.00	0.45	0.47	1.00 (1.00,1.00)	0.14 (0.00,0.30)	1.00	0.07	0.00	1.00	0.00	1.00
Pars triangularis	0.23	0.10	0.00	0.06	0.46	0.41	0.89 (0.64,1.00)	0.19 (0.02,0.35)	1.00	0.35	0.00	0.69	0.00	1.00
Pericalcarine cortex	0.40	0.12	0.04	0.01	0.21	0.23	0.94 (0.82,1.00)	0.34 (0.17,0.50)	0.68	0.35	0.00	0.58	0.00	0.38
Postcentral gyrus	0.32	0.06	0.01	0.03	0.28	0.30	0.94 (0.77,1.00)	0.17 (0.00,0.34)	1.00	0.05	0.00	0.74	0.00	0.83
Posterior-cingulate cortex	0.27	0.00	0.03	0.12	0.44	0.36	0.79 (0.58,0.98)	0.00 (0.00,0.10)	1.00	0.01	0.00	0.10#	0.00	0.64
Precentral gyrus	0.38	0.07	0.03	0.00	0.21	0.23	0.96 (0.87,1.00)	0.24 (0.08,0.40)	1.00	0.01	0.00	0.64	0.00	0.34
Precuneus cortex	0.28	0.12	0.00	0.00	0.18	0.11	1.00 (0.92,1.00)	0.45 (0.33,0.58)	1.00	0.31	0.00	1.00	0.00	1.00
Rostral anterior cingulate cortex	0.24	0.00	0.08	0.08	0.43	0.49	0.74 (0.50,1.00)	0.00 (0.00,0.11)	1.00	0.35	0.00	0.17	0.00	0.19
Rostral middle frontal gyrus	0.26	0.11	0.06	0.00	0.14	0.21	0.90 (0.77,1.00)	0.38 (0.22,0.53)	1.00	0.05	0.00	0.17	0.00	0.07#
Superior frontal gyrus	0.25	0.11	0.06	0.00	0.09	0.16	0.90 (0.78,0.98)	0.48 (0.33,0.61)	1.00	0.01	0.00	0.06#	0.00	0.03#
Superior parietal cortex	0.29	0.08	0.00	0.00	0.23	0.23	1.00 (0.93,1.00)	0.26 (0.11,0.40)	1.00	0.01	0.00	1.00	0.00	1.00
Superior temporal gyrus	0.29	0.06	0.01	0.00	0.18	0.25	0.97 (0.83,1.00)	0.21 (0.04,0.38)	1.00	0.00	0.00	0.91	0.00	0.71
Supramarginal gyrus	0.32	0.06	0.00	0.03	0.23	0.25	0.96 (0.82,1.00)	0.2 (0.03,0.36)	1.00	0.06	0.00	0.77	0.00	1.00

Frontal pole	0.27	0.14	0.00	0.00	0.46	0.48	1.00 (0.77,1.00)	0.23 (0.08,0.38)	1.00	0.34	0.01	1.00	0.00	1.00
Temporal pole	0.25	0.05	0.07	0.01	0.49	0.58	0.87 (0.54,1.00)	0.09 (0.00,0.26)	0.60	1.00[*#]	0.00	0.64	0.00	0.35
Transverse temporal cortex	0.30	0.04	0.06	0.00	0.43	0.49	0.92 (0.73,1.00)	0.09 (0.00,0.24)	0.76	0.91[*#]	0.00	0.71	0.00	0.41
Insula cortex	0.26	0.06	0.06	0.00	0.12	0.13	0.91 (0.81,0.99)	0.33 (0.16,0.49)	1.00	0.00	0.00	0.08[#]	0.00	0.03[#]
**Subcortical Structures**														
Thalamus	0.18	0.10	0.06	0.05	0.17	0.09	0.77 (0.60,0.90)	0.43 (0.27,0.57)	1.00	0.01	0.00	0.00	0.00	0.05[#]
Caudate	0.56	0.05	0.03	0.00	0.07	0.09	0.97 (0.94,1.00)	0.39 (0.22,0.54)	0.90	0.00	0.00	0.15	0.00	0.15[#]
Putamen	0.46	0.08	0.03	0.01	0.23	0.22	0.95 (0.85,1.00)	0.26 (0.09,0.43)	0.98	0.02[#]	0.00	0.67	0.00	0.51
Pallidum	0.24	0.12	0.15	0.01	0.29	0.32	0.77 (0.55,0.92)	0.28 (0.12,0.44)	1.00	0.08	0.00	0.02	0.00	0.01[#]
Hippocampus	0.43	0.09	0.03	0.00	0.15	0.16	0.96 (0.89,1.00)	0.38 (0.21,0.52)	1.00	0.00	0.00	0.56	0.00	0.30
Amygdala	0.29	0.06	0.07	0.00	0.19	0.24	0.90 (0.77,1.00)	0.23 (0.06,0.39)	0.98	0.10	0.00	0.14	0.00	0.05[#]
Accumbens	0.31	0.05	0.02	0.06	0.25	0.22	0.90 (0.74,1.00)	0.19 (0.01,0.36)	0.80	0.39	0.00	0.29	0.00	0.70

Supplementary Table S3. Comparison within three Factor independent pathway models.

IPM1: three factor A, E and C model. Among the three additive genetic A factors, the first A factor was associated with all the eleven ROIs; second A factor was associated with caudate, pallidum, putamen; and the third A factor was associated with temporal, parietal, frontal, and occipital lobes. Similarly three C and E factors were defined. IPM2: in the above IPM1, two of the C-factors were removed and only one common C factor was associated with all the eleven ROIs. IPM3: in the IPM2, the C factor was removed (IPM3; AE model). In all the three models, the eleven ROIs had one additive genetic and one error component as specific factors (only A and E factors).

**Comparison**	**Model**	**-2LL**	**df**	**AIC**	**Δ -2LL**	**Δ df**	**p-value**
	IndPathACE3 (IPM1)	6120.06	3389	-657.94	-	-	-
IPM2 vs IPM1	IndPathACE31C (IMP2)	6126.25	3396	-665.75	6.18	7	0.5186
IPM3 vs IPM1	IndPathAE (IPM3)	6166.72	3407	-647.28	6.66	18	0.0002

Note: The columns are -2LL negative of the twice the log-likelihood; df – degrees of freedom; AIC – Akaike information criteria; Δ -2LL difference in the log-likelihood; Δ df – difference in the degrees of freedom.

Supplementary Table S4. **Comparison of eleven ROI ACE Cholesky and three factor IPM.** ACE1: full ACE Cholesky model. ACE2: full AE Cholesky model. ACE3: CholACERedModel – Cholesky ACE model with only three factors similar to IPM2. ACE4: CholAERedModel – three factor AE model similar to IPM2.

Comparison	Multivariate Models	-2LL	df	AIC	Δ -2LL	Δ df	p-value
	Chol ACE (ACE1)	6035.26	3273	-510.74	-	-	-
ACE2 vs ACE1	Chol AE (ACE2)	6071.69	3339	-606.31	36.43	66	0.9988
ACE3 vs ACE1	Chol ACERedModel (ACE3)	7140.55	3424	-292.55	1105.29	151	<1E-16
ACE4 vs ACE1	CholAERedModel (ACE4)	6917.98	3435	-47.98	882.72	162	<1E-16
ACE2 vs ACE4	CholAERedModel (ACE4)	6917.98	3435	-47.98	846.29	96	<1E-16
ACE1 vs IPM1	IndPathACE3 (IPM1)	6120.06	3389	-657.94	84.8	116	0.9869
ACE1 vs IPM2	IndPathACE31C (IPM2)	6126.25	3396	-665.75	90.98	123	0.9864
ACE1 vs IPM3	IndPathAE (IPM3)	6166.72	3407	-647.28	131.46	134	0.5458

Note: The columns are -2LL negative of the twice the log-likelihood; df – degrees of freedom; AIC – Akaike information criteria; Δ -2LL difference in the log-likelihood; Δ df – difference in the degrees of freedom.

Supplementary Figure S1. Genetic correlations of subcortical structures and cortical ROIs. Genetic correlations were calculated using the volumes of subcortical structures and cortical ROIs (using the bivariate ACE Cholesky model). The colour scale represents the genetic correlations between each region. As this is a symmetric matrix, the cells on the diagonal line represent correlations of a region to itself and are always 1, and were therefore removed. The bar on the right is the colour scale for the correlations. The squares in the figure delineate intra-lobar or intra-subcortical correlations.

Note: Accu=nucleus accumbens; Amyg=amygdala; Caud=caudate; Hipp=hippocampus; Pall=pallidum; Puta=putamen; Thal=thalamus. Frontal ROIs: F1=caudal anterior cingulate; F2=caudal middle frontal; F3=frontal pole; F4=lateral orbitofrontal; F5=medial orbitofrontal; F6=paracentral; F7=parsopercularis; F8=parsorbitalis; F9=parstriangularis; F10=precentral; F11=rostral anterior cingulate; F12=rostral middle frontal; F13=superior frontal. Parietal ROIs: P1==inferior parietal; P2=isthmus cingulate; P3=postcentral; P4=posterior cingulate; P5=precuneus; P6=superior parietal; P7=supramarginal. Temporal ROIs: T1=bankssts; T2=entorhinal; T3=fusiform; T4=inferior temporal; T5=middle temporal; T6=parahippocampal; T7=superior temporal; T8=temporal pole; T9=transverse temporal. Occipital: O1=cuneus; O2=lateral occipital; O3=lingual; O4=pericalcarine. I=insula.

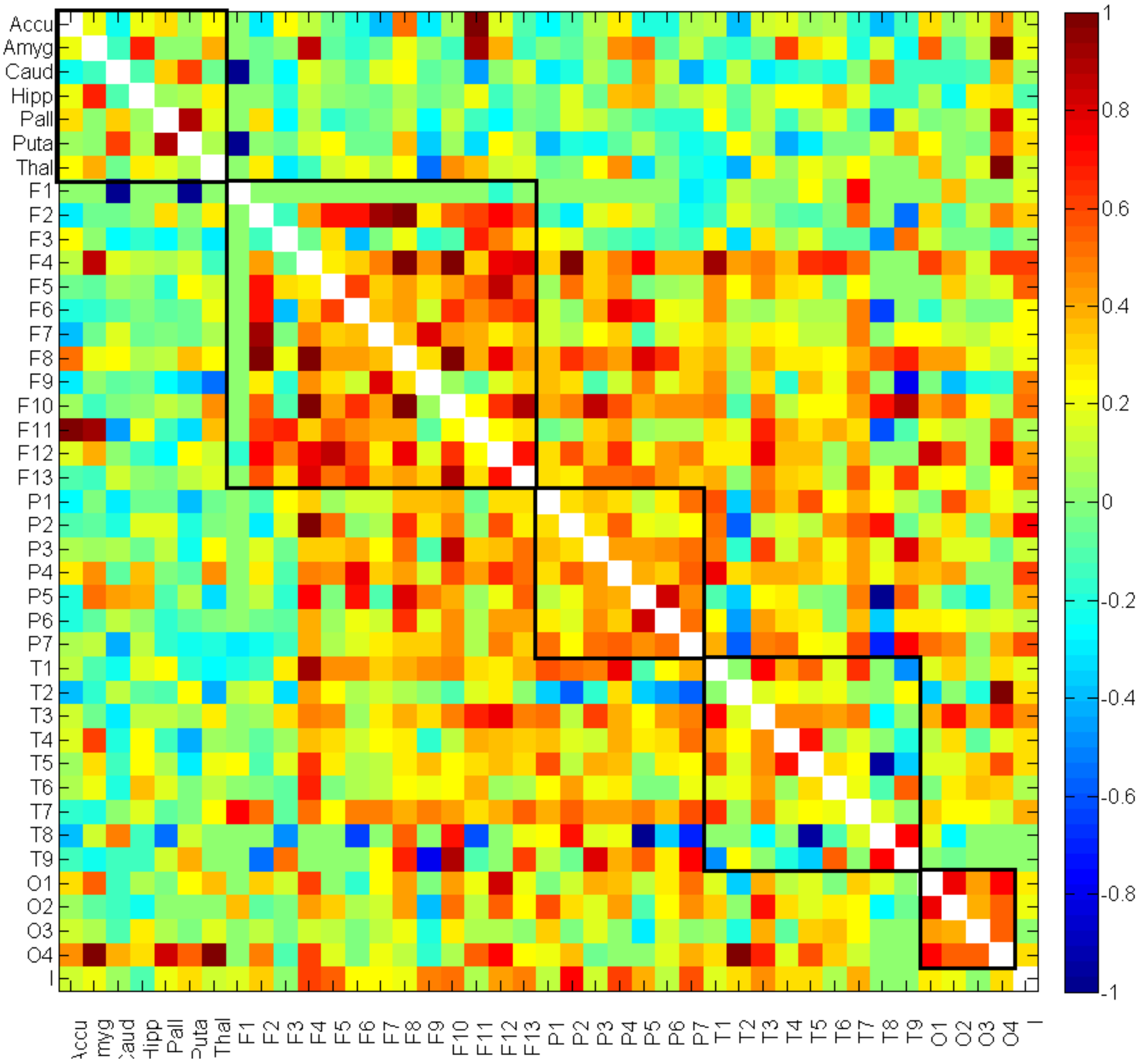

Supplementary Figure S2. Correlation and cluster analysis of 11 ROI multivariate ACE Cholesky model.

(a) Heatmap of genetic correlations between cortical lobes and subcortical volumes using ACE Cholesky model. Dendrogram constructed from hierarchical clustering of (b) the genetic correlation matrix; (c) the phenotype correlation matrix.

Note: ACC = Nucleus accumbens; AMY = Amygdala; HIP = Hippocampus; THA = Thalamus; PAL = Pallidum; PAT = Putatmen; CAU = Caudate; TEMP = Temporal lobe; PARI = Parietal lobe; FRON = Frontal lobe; OCCI = Occipital lobe.

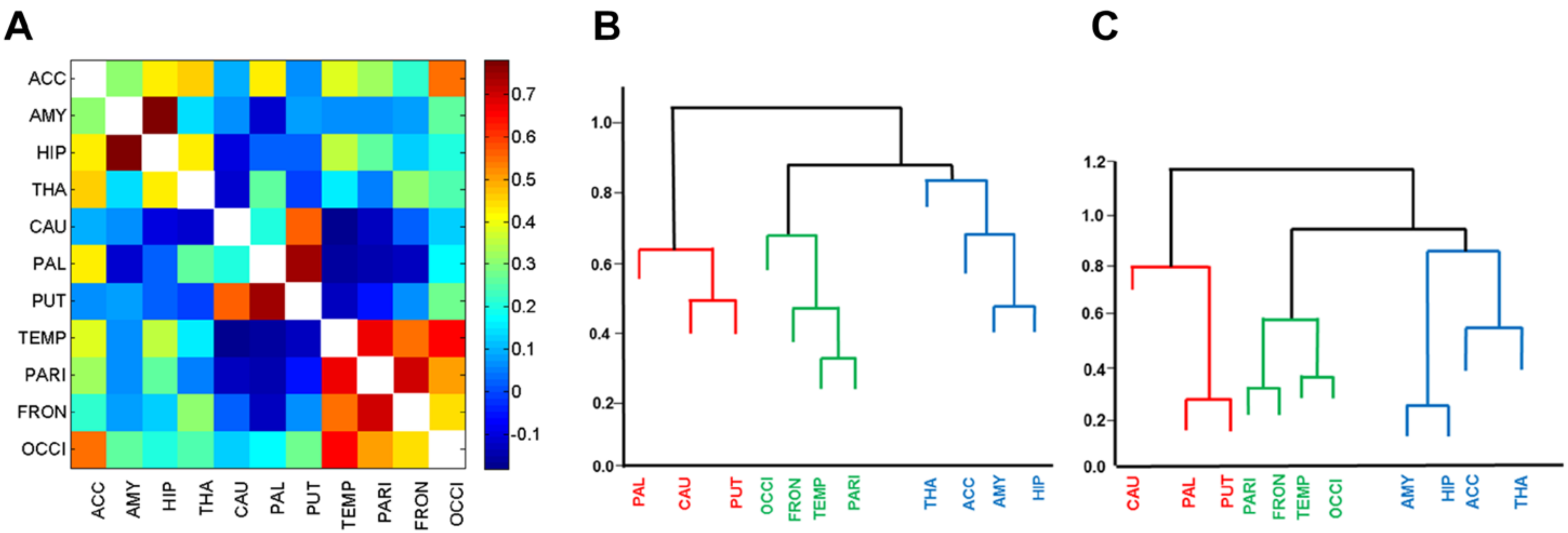

Supplementary Figure S3. Scree plot for the number of factors

The the fa.parallel procedure implemented in R package “psych” (Revelle, W. 2014) was used to determine the number of factors from the scree plot using the phenotype correlation matrix. The dotted lines are based on a normally distributed random data set with 320 samples and 11 variables. The parallel analysis suggests a three factor model for the phenotype correlation matrix.

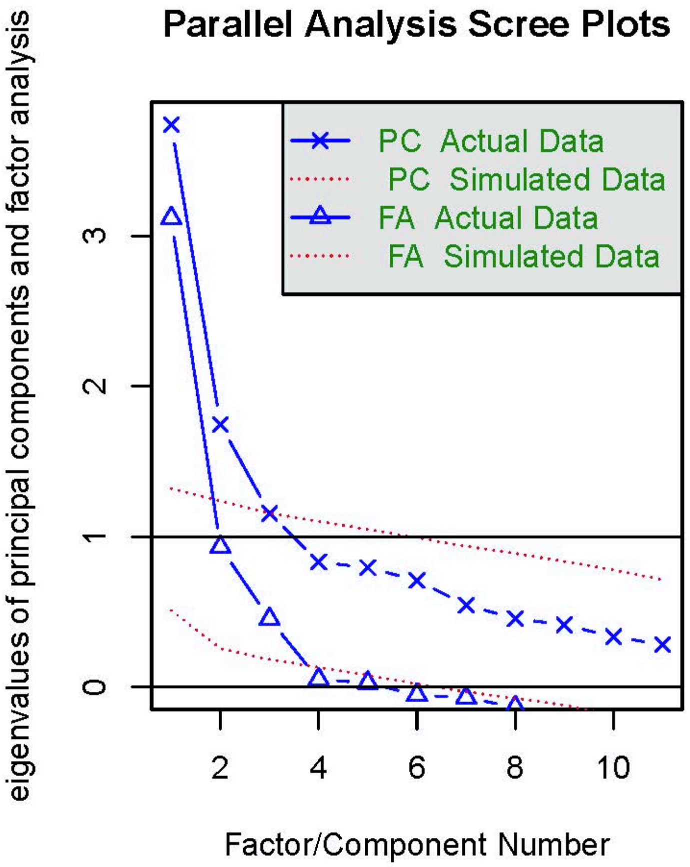

References

Revelle, W. (2014) psych: Procedures for Personality and Psychological Research, Northwestern University, Evanston, Illinois, USA (http://CRAN.R-project.org/package=psych Version = 1.4.8.)

Maechler, M., Rousseeuw, P., Struyf, A., Hubert, M., Hornik, K. (2013). cluster: Cluster Analysis Basics and Extensions. R package version 1.14.4.
